# Supplementary material for: Prevalence and factors associated with human Taenia solium taeniosis and cysticercosis in twelve remote villages of Ranomafana rainforest, Madagascar
Source: PLoS Negl Trop Dis. 2022 Apr 11;16(4):e0010265. doi: 10.1371/journal.pntd.0010265 (PMC9064101; doi:10.1371/journal.pntd.0010265)
Supplement: S1 Fig — Multiple alignments of T. solium Asian or African/American genotypes (T. sol Gen Asia, GeneBank Accession No° AB066488; and T. sol Gen Af/Am, GeneBank Accession No° AB066492.1), T. asiatica (GeneBank Accession No° AB107235.1) and T. saginata (GeneBank Accession No° AB107246.1) were performed. Similarity and variation are marked by a star and space respectively. Common forward and reverse primers are indicated highlighted in bold and with an arrow. (DOCX) [file pntd.0010265.s001.docx]

**Forward primer**

*T. sol* Gen Asia AGTATTTTTAGTTCTATTAATTTTATATGTACATTATATAGAGTTTTTAT**GACTAATATATTTTCTCGTAC**ATCT

*T. sol* Gen Af/Am AGTATTTTTAGTTCTATTAATTTTATATGTACATTATATAGAGTTTTTAT**GACTAATATATTTTCTCGTAC**ATCT

*T. asiatica* AGAATTTTTAGTTCTATTAATTTTATTTGTACTTTATATAGAATATTTAT**GACTAATATATTTTCTCGTAC**TTCT

*T. saginata* AGAATTTTTAGTTCTATTAATTTTATTTGTACTTTGTATAGAATATTTAT**GACTAATATATTTTCTCGTAC**TTCT

** *********************** ***** ** ****** * ************************** ***

*T. sol* Gen Asia ATAGTGTTATGATCTTATTTATTTACATCTATCTTGTTATTGGTTACTTTACCTGTTTTGGCAGCCGCTGTTACT

*T. sol* Gen Af/Am ATAGTGTTATGATCTTATTTATTTACATCTATCTTGTTATTGGTTACTTTACCTGTTTTGGCAGCCGCTGTTACT

*T. asiatica* ATAATATTATGGGCTTATTTATTTACGTCAATCTTATTGTTAGTTACTCTTCCTGTGTTAGCAGCTGCTATTACT

*T. saginata* ATAATATTGTGGGCTTATTTATTTACGTCTATTTTATTATTAGTTACTCTTCCTGTATTAGCAGCTGCTATCACT

*** * ** ** ************* ** ** ** ** ** ****** * ***** ** ***** *** * ***

*T. sol* Gen Asia ATGCTTCTATTTGATCGTAAATTTAGTTCTGCGTTTTTTGATCCATTAGGAGGTGGTGATCCTGTTTTGTTTCAA

*T. sol* Gen Af/Am ATGCTTCTATTTGATCGTAAATTTAGTTCTGCGTTTTTTGATCCGTTAGGAGGTGGTGATCCTGTTTTATTTCAA

*T. asiatica* ATGCTTTTATTTGATCGTAAATTTAGTTCTGCGTTTTTTGATCCGTTAGGTGGTGGTGATCCTGTTTTATTTCAA

*T. saginata* ATGCTTTTATTTGACCGTAAATTTAGTTCTGCGTTTTTTGATCCATTGGGTGGTGGTGATCCTGTTTTATTTCAA

****** ******* ***************************** ** ** ***************** ******

*T. sol* Gen Asia CATATGTTTTGATTTTTTGGTCATCCCGAGGTTTATGTGTTAATTCTTCCGGGGTTTGGTATAATTAGTCATATA

*T. sol* Gen Af/Am CATATGTTTTGATTTTTTGGTCATCCTGAGGTTTATGTGTTAATTCTTCCGGGGTTTGGTATAATTAGTCATATA

*T. asiatica* CATATGTTTTGATTTTTTGGTCATCCGGAGGTTTATGTTTTAATTATTCCTGGTTTTGGTATGATTAGTCATATA

*T. saginata* CATATGTTTTGATTTTTTGGTCATCCAGAGGTTTATGTTTTAATTATTCCTGGTTTTGGTATGATTAGTCATATA

************************** *********** ****** **** ** ******** ************

*T. sol* Gen Asia TGTTTGAGTATAAGTATGTGTTCTGATGCTTTTGGCTTTTATGGGTTATTGTTTGCTATGTTTTCAATAGTATGT

*T. sol* Gen Af/Am TGTTTGAGTATAAGTATGTGTTCTGATGCTTTTGGCTTTTATGGGTTATTGTTTGCTATGTTTTCAATAGTATGT

*T. asiatica* TGTTTAAGAATAAGTATGTGTCCGGATGCTTTTGGTTTTTATGGTTTGTTATTTGCTATGTTTTCAATAGTATGT

*T. saginata* TGTTTAAGAATAAGTATGTGTCCAGATGCTTTTGGTTTTTATGGTTTGTTGTTTGCTATGTTTTCAATAGTGTGT

***** ** ************ * *********** ******** ** ** ******************** ***

*T. sol* Gen Asia TTAGGAAGAAGTGTGTGAGGACATCATATGTTTACGGTTGGGTTAGATGTTAAGACGGCTGTATTTTTTAGTTCT

*T. sol* Gen Af/Am TTAGGAAGAAGTGTATGAGGGCATCATATGTTTACGGTTGGGTTAGATGTTAAGACGGCTGTATTTTTTAGTTCT

*T. asiatica* TTGGGGAGAAGTGTGTGGGGTCATCATATGTTTACGGTTGGATTAGATGTTAAGACTACTGTGTTTTTTAGTTCG

*T. saginata* TTGGGGAGAAGTGTGTGGGGTCATCATATGTTTACGGTTGGGTTAGATGTTAAGACTGCTGTGTTTTTTAGTTCG

** ** ******** ** ** ******************** ************** **** ***********

*T. sol* Gen Asia GTTACTATGATAATTGGAGTGCCTACGGGGATTAAGGTTTTTACTTGGCTTTATATGCTTTTAAAATCTCGTGTT

*T. sol* Gen Af/Am GTTACTATGATAATTGGAGTGCCTACGGGGATTAAGGTTTTTACTTGGCTTTATATGCTTTTAAAATCTCGTGTT

*T. asiatica* GTTACTATGATAATAGGAGTACCAACAGGAATAAAGGTTTTTACTTGACTTTATATGCTTTTAAATTCTCGTGTA

*T. saginata* GTTACTATGATAATAGGAGTACCAACAGGAATAAAGGTTTTTACTTGACTTTATATGCTTTTAAATTCTCGTGTA

************** ***** ** ** ** ** ************** ***************** ********

*T. sol* Gen Asia AATAAGAGTGATCCGGTTTTATGATGAATAATTTCGTTTATAGTATTGTTTACATTTGGTGGTGTAACCGGTATT

*T. sol* Gen Af/Am AATAAGAGTGATCCGGTTTTATGATGAATAATTTCGTTTATAGTATTGTTTACATTTGGTGGTGTAACTGGTATT

*T. asiatica* AATAAGAGGGATCCTATATTGTGGTGGATAGTTTCTTTTATAGTGTTGTTTACCTTTGGTGGTGTGACTGGTATT

*T. saginata* AATAAGAGTGATCCTATATTGTGGTGAATAGTTTCTTTTATAGTGTTGTTTACTTTTGGTGGTGTGACTGGTATT

******** ***** * ** ** ** *** **** ******** ******** *********** ** ******

**Reverse primer**

*T. sol* Gen Asia ATTCTATCTGCTTGTGTATTAGATAAAGTTCTTCATGATACTTGGTTTGTTGTTGCT**CATTTTCATTATGTTATG**

*T. sol* Gen Af/Am ATTCTATCTGCTTGTGTATTAGATAAAGTTCTTCATGATACTTGGTTTGTTGTTGCT**CATTTTCATTATGTTATG**

*T. asiatica* GTGTTGTCTGCTTGTGTATTGGATAAAGTTTTGCATGATACTTGATTTGTTGTTGCC**CATTTTCATTATGTTATG**

*T. saginata* GTGTTGTCTGCTTGCGTATTGGATAAAGTTTTGCATGATACTTGATTTGTTGTTGCT**CATTTTCATTATGTTATG**

* * ******** ***** ********* * *********** *********** ******************

*T. sol* Gen Asia **TC**ATTAGGGTCTTATATCAGTATAATAATTATGTTTGTTTGGTGG

*T. sol* Gen Af/Am **TC**ATTAGGGTCTTATATTAGTATAATAATTATGTTTGTTTGGTGG

*T. asiatica* **TC**GTTAGGGTCTTATATAAGAATAATAATTATGTTTATTTGATGG

*T. saginata* **TC**ATTAGGGTCTTATATAAGAATAATAATTATGTTTATTTGATGG

** ************** ** *************** **** ***
