# Supplementary figures and images for: Prevalence and factors associated with human Taenia solium taeniosis and cysticercosis in twelve remote villages of Ranomafana rainforest, Madagascar
Source: PLoS Negl Trop Dis. 2022 Apr 11;16(4):e0010265. doi: 10.1371/journal.pntd.0010265 (PMC9064101; doi:10.1371/journal.pntd.0010265)

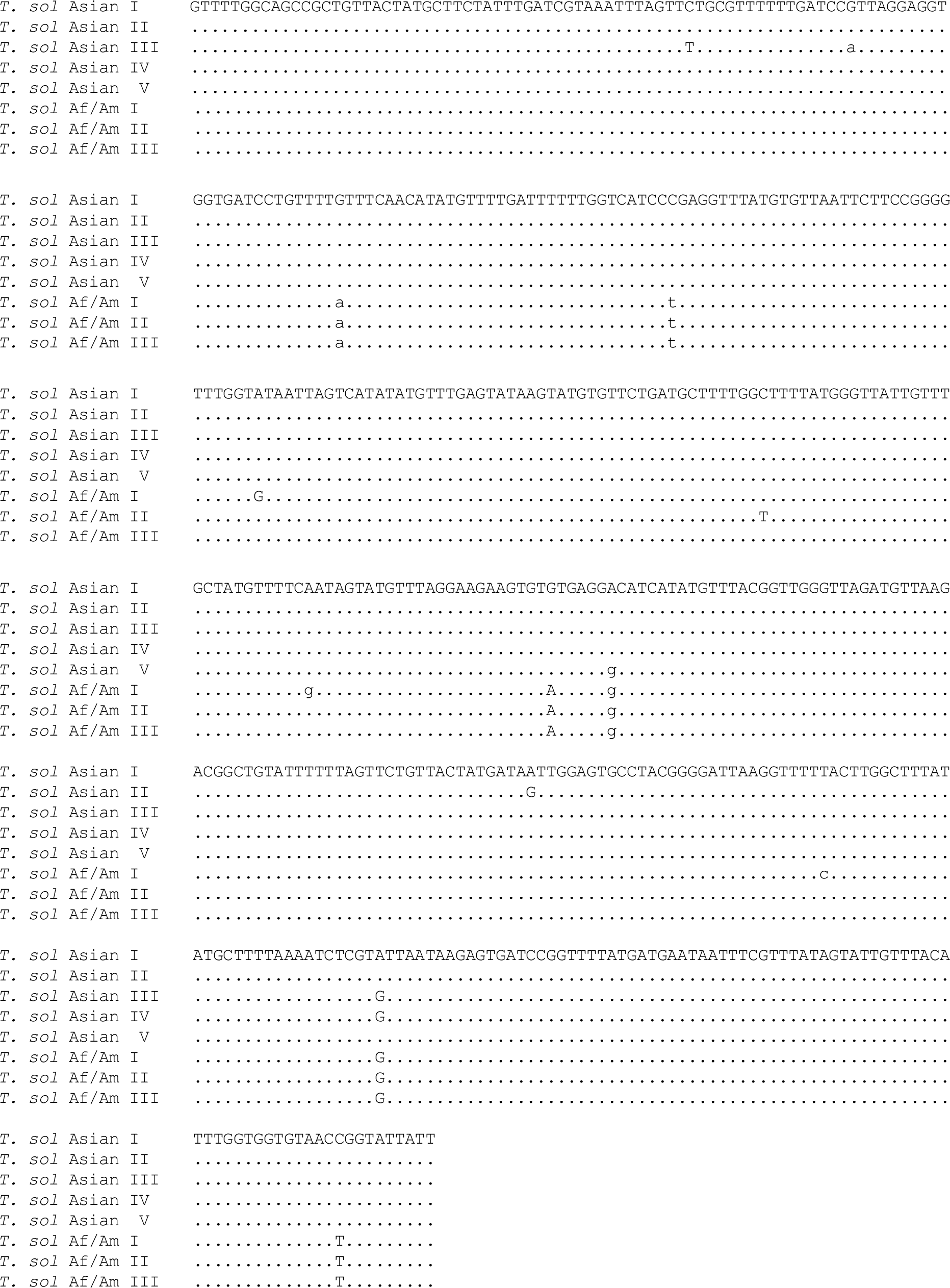

Supplement: S2 Fig — Five T. solium Asian genotypes (T. sol Asian I to V) and three for T. solium African-American genotype (T. sol Af/Am I to III) were aligned. T. solium Asian genotype cluster I sequence is shown as reference. Similarity is indicated with dot. Variant nucleotides are shown in lowercase and in capital letters if affecting amino acid sequence. Comparing to the Asian T. solium genotype cluster I (containing the majority of sequences obtained in this study), the Asian genotype cluster II (from Toamasina, Madagascar) showed one changed base (G instead of A) at the position 934 of the cox 1 completed sequence which changed the corresponding amino acid while the Asian genotype cluster III (from Indonesia) presented 3 substituted nucleotides (C, G and A changed into T, A and G: at the positions 650, 666 and 994 respectively). This last changed base was also the only variation observed in the Asian genotype cluster IV (from Nepal, Japan, China and Korea). The last Asian T. solium genotype obtained in this study, from Sahavoemba (Asian genotype cluster V) was also closely related to the majority of Asian T. solium genotype found in this study (Cluster I) except for one base: A modified in G at the position 867 which is a common substitution in the African-American T. solium genotype. The only African/American genotype found in this study forming the African-American genotype cluster III (with Tanzania, Toliara Madagascar and Mexico) showed six substituted nucleotides (of which 3 modified amino acids) compared to the Asian genotype cluster II. The African-American genotype cluster II (Ecuador) and I (Mexico, Cameroon and Brazil) counted seven and nine substitutions respectively both with four changed amino acids. (TIF) [file pntd.0010265.s002.tif]
