# Supplementary material for: Prevalence and factors associated with human Taenia solium taeniosis and cysticercosis in twelve remote villages of Ranomafana rainforest, Madagascar
Source: PLoS Negl Trop Dis. 2022 Apr 11;16(4):e0010265. doi: 10.1371/journal.pntd.0010265 (PMC9064101; doi:10.1371/journal.pntd.0010265)
Supplement: S1 Table — The geographic coordinates (GPS) of the 12 villages are indicated. (DOCX) [file pntd.0010265.s003.docx]

| Townships | Fokontany | Villages | Latitude | Longitude |
| --- | --- | --- | --- | --- |
| Ranomafana | Tsaramandroso | Ampitavanana | 21°14'55.52"S | 47°30'22.44"E |
|  |  | Bevoahazo | 21°12'32.19"S | 47°29'54.99"E |
|  |  | Torotosy | 21°13'34.17"S | 47°30'22.28"E |
|  | Vohimarina | Ambinanindranofotaka | 21°23'30.408"S | 47°26'16.404"E |
|  |  | Mangevo | 21°22'58.98"S | 47°28'35.039"E |
|  |  | Marojano | 21°22'14.232"S | 47°28'53.544"E |
|  |  | Sahavanana | 21°22'18.48"S | 47°29'44.987"E |
|  |  | Sahavoemba | 21°20'56.796"S | 47°30'4.932"E |
| Kelilalina | Ampitambe | Ampitambe | 21°15'50.86"S | 47°34'51.92"E |
|  | Mandrivany | Ankazotsara | 21°16'08.17"S | 47°34'29.35"E |
|  |  | Mandrivany | 21°14'57.56"S | 47°33'39.62"E |
|  | Kianjanomby | Kianjanomby | 21°14'20.84"S | 47°34'27.95"E |

Coordinates are given in the WGS 84 coordinate reference system (in degrees and decimal minutes, DMS).
