# Supplementary material for: Prevalence and factors associated with human Taenia solium taeniosis and cysticercosis in twelve remote villages of Ranomafana rainforest, Madagascar
Source: PLoS Negl Trop Dis. 2022 Apr 11;16(4):e0010265. doi: 10.1371/journal.pntd.0010265 (PMC9064101; doi:10.1371/journal.pntd.0010265)
Supplement: Attachment — Submitted filename: Response_to_reviewers.docx [file pntd.0010265.s004.docx]

Madagascar, August 24^th^, 2021

**Jessica K Fairley, MD, MPH**

Associate Editor

**Aaron Jex**

Deputy Editor

PLOS Neglected Tropical Diseases Journal

Manuscript Number: PNTD-D-21-00272R1

Dear Editors,

We thank you for reviewing our submission entitled “Prevalence and factors associated with human Taenia solium taeniosis and cysticercosis in twelve remote villages of Ranomafana rainforest, Madagascar”, PNTD-D-21-00272R1. We are grateful both to the editorial team and to the reviewers for their considerate feedback. By this letter, we wish to submit a revised version for publication as a major article in the *PLOS Neglected Tropical Diseases.* We thank the reviewers for their insightful comments, and we believe that their comments have helped strengthen this paper. Below please find point-by-point responses to the reviewers’ comments that we have written in blue.

Sincerely,

Anjanirina RAHANTAMALALA

************************************************************

**Reviewer's Responses to Questions - Manuscript: PNTD-D-21-00272R1**

Key Review Criteria Required for Acceptance?

As you describe the new analyses required for acceptance, please consider the following:

Methods

-Are the objectives of the study clearly articulated with a clear testable hypothesis stated?

-Is the study design appropriate to address the stated objectives?

-Is the population clearly described and appropriate for the hypothesis being tested?

-Is the sample size sufficient to ensure adequate power to address the hypothesis being tested?

-Were correct statistical analysis used to support conclusions?

-Are there concerns about ethical or regulatory requirements being met?

**Reviewer #1:** The objectives are clearly stated and the study design seems appropriate, but some important details are still missing (see below). In general, the analyses seem appropriate and ethical and regulatory requirements are met.

1-L157: how exactly were the households randomized? And out of how many households were these households randomly selected (i.e. how many were not selected)? And why 164? Considering the 574 initially fixed. A sample size consideration is missing.

We thank the reviewer for this insightful comment.

For the cysticercosis seroprevalence study and the associated risk factor analysis, the sample size was calculated considering a predicted prevalence of 7% [Ministry of Public Health. Plan directeur de lutte contre les Maladies Tropicales Negligées - (MTN) 2016 - 2020. 2015]. With a prespecified confidence interval of 90% to support a significant difference with alpha = 0.05, and an absolute error of 2%, the expected total sample size was estimated at 440 people (see table below in explanation n°5).

The 164 households (among 1104 total households identified in the 12 villages targeted) were selected using an electronic random number generator on the fokontany census in 2016 and corresponding to 721 villagers ≥ 5 years of old. This information was completed in the L157 in the original paper which becomes L163 in the modified version. **The final number of households investigated was 150, corresponding to 543 willing participants. The census and the number of households per village are detailed in the Excel file called "database_IFA1", sheet “Households”.**

2-L196: please give an indication of the time of conservation (between stool sample collection and extraction/PCR analysis):

Sampling was carried out between June and August 2016 and DNA extractions were performed in October 2016. Extracted DNAs were correctly stored at -20°C until used. The first round of PCR analysis was realized in November 2016 and validated with a second round of PCR/sequencing analysis in August 2017. Indeed, the PCR assay initially used was done with the primer sets described by Yamasaki et al., for genus identification and to differentiate the 2 genotypes of *T. solium*. However, our data shown that these primers were cross-reacting between Taenia species. Finally, we used a common Taenia genius primer pair to realize the PCR and thereafter, sequenced all the amplicons obtained.

3- L188-193: The questionnaire should be described more clearly. The results of the questions could be very different, depending on how the questions were formulated. For example the question pork consumption (yes/no) is too vague. It could be interpreted as having ever eaten pork in your life, but also as eating pork weakly, which are two completely different things. I would suggest to add the questionnaire in appendix.

The question about the pork consumption was “How often do you eat pork a year?”. The whole questionnaire was added in appendix.

4-Is having more than once headache every 3 months considered as chronic headache? It would be good to add a reference to this definition of chronic headache.

Effectively, the term “chronic” is not adapted by referring to the definition proposed by the International headache society. Also we propose to replace with “frequent”. The corresponding modification was done in the L390, in Table 1, in L521 and in Table 4 in the modified version.

**Reviewer #2:** The hypothesis is clearly stated as the determination of the prevalence of human *Taenia solium*, taeniasis/cysticercosis and risk factors in remote areas of Madagascar. The human population of the area is clearly described. The study design is appropriated and the sample size seems sufficient to address this hypothesis, statistical analyses do not rise any concern. Ethical and regulatory requirement seem to have been met.

**Reviewer #3:** The objectives of the study are clearly articulated and the population is also clearly described. The sample size is sufficient to ensure adequate power to address the hypothesis. The statistical analysis support conclusions.

--------------------

<b>Results</b></br></br>

-Does the analysis presented match the analysis plan?</br>

-Are the results clearly and completely presented?</br>

-Are the figures (Tables, Images) of sufficient quality for clarity?</br></br>

Reviewer #1: The analyses match the analysis plan and the results are compete, but at times the results are over interpreted (especially regarding the results at village level).

5-The primary aim and secondary/exploratory outcomes could be defined more clearly. I think the sample size is not appropriate for a thorough risk factor analysis. In my opinion, the prevalence estimates seem like the primary aim, and the risk factors are more exploratory. I would suggest to make that more clear.

Taking into account the calculation of the sample size in the explanation n°1 and according to table below, we think that the final sample size (459 participants) with a confidence interval between 90% and 91% is still correct for the analysis of risk factors.

| **Confidence level (%)** | **Predicted prevalence** | **Absolute error** | **Sample size** |
| --- | --- | --- | --- |
| 99 | 0,07 | 0,02 | 1080 |
| 98 | 0,07 | 0,02 | 881 |
| 97 | 0,07 | 0,02 | 766 |
| 96 | 0,07 | 0,02 | 686 |
| 95 | 0,07 | 0,02 | 625 |
| 94 | 0,07 | 0,02 | 576 |
| 93 | 0,07 | 0,02 | 534 |
| 92 | 0,07 | 0,02 | 499 |
| 91 | 0,07 | 0,02 | 468 |
| 90 | 0,07 | 0,02 | 440 |

6-L348: 574 willing participants, out of how many in these households? Do you have an indication how many people in the selected households were not willing to participate? And L350: not really out of 574 villagers, but out of 574 participants. I assume that the number of villagers is higher than this number because of the exclusion criteria (above 5) and willingness to participate. If available, it would be good to provide this information in Figure 2 and/or the text (how many were excluded because of age or because they were not willing).

We have modified and detailed in Figure 2 the detail about the selected household as following: “164 selected household corresponding to 721 villagers ≥5years of old (out of 4767 total villagers in the 12 villages investigated). 107 villagers (<5 years of old) were not willing to participate”. This information was also considered in the modified version in L359-360.

7-L389: this is an over-interpretation of the data; I don’t think you should conclude these villages are “most infected” based on the data (because it’s based on 0, 1 or 2 positive cases per village). This is also clear from the wide CI around the estimates in Table 2. The % positive samples per village should therefore not have digits, because the estimates are very uncertain due to the relatively low sample size per village. It’s OK to present the results per village, but don’t overanalyse them. As mentioned earlier, it would be good to give an indication of the number of people living in each village, because that’s not clear from the manuscript. For example the results from only 10 people from Bevoahazo: how many people actually lived in that village at the time of recruitment?

We thank you for these very important remarks, details of the total number of villagers per village, and the total number of villagers >5 years old per village corresponding to the selected household have been added in tables 2 and 3. The number of Taenia carriers was only presented per village without comparison and taken into account in L411-414.

8-L460-463: I find this sentence very difficult to follow. And also for the sentence below: the message is not very clear, and as mentioned above, the categorization of villages between presence/absence of taeniosis based on the very few positive samples is likely an overinterpretation of the results due to the large uncertainty around the taeniosis prevalence estimates per village.

The sentence between L460-465 was changed in the L478-480 of the modified version as following “Depending on the village investigated, the exposure rate found by Ab-ELISA and IgG Western Blot assays ranged between 12.5% - 50% and between 16.2% - 69.6% respectively (Table 3)”

The seroprevalence per village was indicated without comparison between presence/absence of taeniosis cases.

9-L466: the sample size is too low to draw conclusions about which village has the highest prevalence (Marojano is only based on 23 samples)

The comparison of prevalence of taeniosis between villages has been removed from the original text.

10-L485-489: same comment as above

The comparison of the presence/absence Taenia carrier between villages for the seroprevalence of circulating antigens has also been removed from the original text.

11-L500: how was this cutoff (4 or more) determined?

The cuttoff (4 or more individuals) for the household was determined by the average size of household in Madagascar, which was around 4 since 2005 (Priority household survey, Instat, 2000). The last population census in 2018 reported an average size of 4.2 individuals per household, and 4.3 in rural areas (third General Population and Housing Census, RGPH-3).

12-Same comment for house distance.

A part of the results of this investigation already published (Hakami et al., 2019: doi: 10.1080/20477724.2019.1589927) and concerning other intestinal parasites (soil transmitted helminth STH and Strongyloides stercoralis) had reported that individuals infected by any parasite were significantly found to be living further away from the main road (mean: 6.3 km) than those not infected (mean: 4.5 km) (p = 0.002). A positive correlation between having a STH infection and living further from the main road (R2 = 0.57) was also found.

13- First box with Excluded: “missing epidemiological data”: what exactly does this mean? In table 1, it seems there are missing data for some of the 459 participants (but they are not excluded)? It would be better to specify how many are missing due to missing epidemiological data (and what exactly is meant) and how many due to missing biological samples (and which: serum and/or stool). Second box with Excluded: all serological analyses (not analysis)

The information about missing data was added into the Figure 2 and in the results section in the modified text L360-366: **i)** Missing basic epidemiological data: gender and age were missing (5 for the final sample size, many people do not remember their age and do not have birth certificate in these remote areas which counts 121 for the total number of the 12 villages). **ii)** Missing epidemiological data for statistical analysis: when the completion of variables considered in the manuscript was less than 25% (for 11 participants). **iii)** Missing biological samples: when the blood could not be collected for various reasons (very mobile or too thin veins or fear, etc.) or the participants did not return the stool samples (Stool: 2, serum: 24). **iv)** Insufficient serum to perform all serological analysis: sampling was carried out by capillary and using microvette with a maximum volume of 500µL which was too limited for 73 participants.

Reviewer #2: Analyses presented match the analysis plan and the results are clearly presented.

Reviewer #3: The results and figures are clearly and completely presented. The figures are of sufficient quality for clarity.

--------------------

<b>Conclusions</b></br></br>

-Are the conclusions supported by the data presented?</br>

-Are the limitations of analysis clearly described?</br>

-Do the authors discuss how these data can be helpful to advance our understanding of the topic under study?</br>

-Is public health relevance addressed?</br></br>

Reviewer #1: The discussion clearly addresses the public health relevance and also includes some limitations of the study. The discussion could be slightly more comprehensive.

14-L540-550: this whole section seems rather introduction than discussion. The results from the current manuscript are currently not mentioned/discussed here.

This part L540-550 of the original version has been moved and compiled to the introduction section into the L121-125 and L127-128 in the modified text.

15- L582: “more prevalent in adults”: this cannot be concluded from the data with certainty: the difference between adults and children was not significant and actually seemed very minor. The CI are almost completely overlapping. This should be rephrased; perhaps include other studies where they did find significant differences with age.

Effectively, comparison of the prevalence of taeniosis between adults and children was removed from the original text.

L600: I don’t understand the “common history” between pigs and human.

This “common history” that we wish to be more explicit in the modified text was moved in the end of the paragraph about genotyping and rephrased in L622-627 as following “This phylogenetic characterization enables us to hypothesize that the definitive and the secondary hosts (human and pig) could share common infestation history. Indeed this sympatric distribution of the two genotypes and the predominance of the Asian genotype could be reflected and conserved in both hosts and corresponding to the dual origin (Asian and African) of the Malagasy people”.

L645-647: “water source”: it’s not so clear to which results this is referring to: it seems that river water is protective according to Table 5 (relative to stand pipe). Also, why is water source not included in the initial model in table 6 since p<0.2 according to table 5?

This ambiguity was rectified and explained in the modified text in L660-662 “The risk analysis obtained with the variable “house water source” using stand pipe and the probable protective factor when using the river with renewed running water seems to strengthen this previous report”. The variable "house water source" has been also included in the initial model of Table 6. This protective effect of the river as a source of water was also confirmed in the final model and interpreted in the L540.

L675: I don’t think any results/analyses at household level are included in the paper?

Analysis of the *T. solium* infections within the same household (where a Taenia carrier was detected) was completed in Table 3 and interpreted in paragraph corresponding to each serological test result in L480-483 for the antibodies analysis and in L510-511 for the antigens analysis. Risk factor associated to the number of household members was also analyzed and seems to be significantly associated with *T. solium* infections.

Reviewer #2: Conclusions are supported by the data presented, but as Taenisasis/Cysticercosis is also a zoonose. This study rise even more question to address this public health problem. Beyond determination of prevalence in remote area, this work focus upon genetic of taenia solium and lack of perspective in order to give valuable data to fight taenia solium in this area and in low and middle income countries in general diagnosis and treatment of teania carrier is one of the . Strains of taenia solium have been clearly identified and genotyped, but neither characteristic linked to the intermediate hosts (pork) nor the neurological conditions or pathogenicity (burden) in human participants been evaluated. Moreover, more information upon local habits and environment could help to explain the results of multivariate analyses.

An extensive questionnaire in terms of neurological conditions was drawn up in consultation with hospital neurologists, which unfortunately could not be properly exploited due to a large proportion of the participants with lacked data (76.3%). Indeed these questions associated with epilepsy remain a taboo and shameful subject, especially in those isolated areas. These precise questions and detailed questions took time to answer.

Nevertheless important criteria of health burden associated with neurocysticercosis according to the classification of Del Bruto and collegues in 2017 (such as: specific anticysticercal antibodies/cysticercal antigens detected by immunodiagnostic tests, evidence of a household contact with *T. solium* infection, and clinical manifestations suggestive of neurocysticercosis: mainly seizures starting in individuals aged 20–49 years) were noted during this study. More information about local habits and environment has been added in Table 1 which was initially lightened so as not to overcharge the publication.

Concerning the pig, some questions about pork consumption, the source of pork and the breeding/presence of pork close to participants' habitats were investigated in this study. Unfortunately swine cysticercosis was not investigated during this study. However other study in 2017 to continuing this study and targeting one of the 12 villages investigated by this study made it possible to better respect the “one health” approach and complete the control strategies. Indeed, mass drug administration (MDA) for the whole population of all ages >5 years old, investigation in some pigs and in main water source points were carried out with a follow-up before and after the MDA.

As an example, during deworming campaigns, taenia carriers are expected to expel scolex and proglottids, the latter need to be correctly treated in order to be sure to efficiently destroy eggs and avoid a massive contamination of environment and particularly the water used and ingested by both humans and livestock. Inefficient treatment of gravid proglottids following mass treatment can lead to a counter intuitive augmentation of cases in area. Identification of tapeworm carriers remains a challenging problem in community and anti-helminthic treatment could help in such.

As mentioned in L158-162 in the modified text of the methods section, to recover the whole teania worm, the following treatment was done for each Taenia carrier: niclosamide: 2gr orally single dose + oral purgative: before and after niclosamide, which was suitably realized to recover the whole worm properly. A follow-up before, during and after the treatment during 6 days was carried out for each patient. To avoid dissemination of eggs in the environment, these patients defecated in a plastic bag (Well-preserved in a container) during 3 days after the anti-parasitic treatment.

Authors broach the subject of self-infestation (Cysticercosis) in tapeworms carriers but diagnostic of infections are not presented for the 11 carriers identified in the study nor for the people living in the same household while a higher rate of exposure and infection are expected.

We have added the serological results (Ab-ELISA, EITB and Ag-ELISA) of each Taenia carrier and all household contacts among *Taenia* carrier in Table 3. Corresponding texts were explained in L695-698.

Reviewer #3: The conclusions are supported by the data presented and limitations of analysis are clearly described. Data are public health relevance addressed.

--------------------

<b>Editorial and Data Presentation Modifications?</b></br><br/> Use this section for editorial suggestions as well as relatively minor modifications of existing data that would enhance clarity. If the only modifications needed are minor and/or editorial, you may wish to recommend “Minor Revision” or “Accept”.

Reviewer #1: Author summary: I’m not sure if this is understandable enough for the lay public

The author summary was modified by removing parts detailing the life cycle of T. solium.

L86: associated “with”? .

Becoming “associated with” in the modified text L87

L92-93: the life cycle is not maintained among humans. This is confusing and should be rephrased

Effectively, infestation from human to human is more suitable. We prefer to remove “among human” to avoid ambiguities.

L139-141: this sentence is not clear to me. Perhaps explain fokontany already here (first use of the word).

The sentence was rephrased in L141-144 as following “In 2015, national mapping program reported 0% of taeniosis prevalence in 14 fokontany (the smallest administrative subdivision of Madagascar) including 21 rural villages (with one village targeted by this study)”

L179: common instead of commonly

This has been rectified in L187 in the modified text

L194: “spp.” should not be in italics (a species name should be in italics, but the word species not)

This has been rectified in L202 in the modified text

L304: use sensitivity (not sensibility)

This has been rectified in L315 in the modified text

L341: this sentence is confusing as it reads “p > 0.05 as statistically significant”, whereas values < 0.05 are considered statistically significant. It should be rewritten

This has been rectified in L352 in the modified text

L352: diagnosis instead of diagnostic?

This has been rectified in L365 in the modified text

Table 1: why not just write “4 to 6” instead of “4 ≤ pers. ≤ 6”?

This has been rectified in Table 1

L405-409: I think this is material and methods (or introduction), but not results Fig 3 caption: not really “from Taenia eggs of human stool”, but rather DNA extraction of stool samples in which eggs were detected? Based on M&M, it seems that DNA was extracted from the stool sample, not only the eggs? Same for L429

This sentence between L405-409 in the original text was compiled and moved to the introduction section in L231-235.

“from Taenia eggs of human stool” has been rectified in the legend of the Fig 3 in L441 as following “from human stool *with* detected *Taenia* eggs”.

L479: cysticerci (spelling mistake)

« cysticerci » has been removed and the sentence was rephrased in L498-499 as following « Current cysticercosis was detected by measuring the excretory/secretory circulating *T. solium* antigens based on B158/B60 enzyme-linked immunosorbent assay (Ag-ELISA)”

L496 “behaviors factors” (grammar) and L497: Table (not tables)

This was rectified and modified in L514-515 of the modified text as following “Several epidemiological and **behavioral** factors were evaluated for their association with *T. solium* infections and summarized in **Table 4.”**

L499: these are methods (not results)

This sentence has been removed from the text as the p-value<0.2 included in multivariate analysis was already precise in methods.

L501: school attendance = education? I would suggest to use the same wording as in table 4. I don’t understand the * for education in Table 4, since the p value is > 0.2. I assume it’s a mistake?

Effectively “education” was not significant and “School attendance” was removed in the modified text.

Table 4: there seem to be many more questions compared to Table 1? Why are some of these questions also included in Table 1, but not others?

Effectively some variables (pig nearby, meat source, house distance to the national main road, house water source, soap use for washing hands, cysticercosis knowledge, and age of first seizure) have initially removed to the global description of the study population in Table 1 to not overload the publication. These variables had however considered for the risk factor analysis. These variables have been added in Table 1.

L508-509: This is confusing, since this seems to be a mixture of both EITB and Ag ELISA results?

This sentence was removed from the original text and was entirely rephrased by listing the significant factors with a p≤0.05 and the variables included in the multivariate analysis with a p <0.2, as modified in L515-528

L538: grammar

“analyzing factors associated in rural and remotely region of Madagascar” was modified and rephrased with “and analyzing the associated risk factors in rural and remotely region of Madagascar” in L558

L545: ranging between what? Between villages/districts/…?

Sentences between L541-550 were removed from the original text as these previous studies were already mentioned in the introduction section.

L557-565: this is a long and complicated sentence. Please rephrase it. Also, I agree that you should only compare prevalence estimates among studies with a comparable methodology and that microscopy might result in a lower prevalence estimate, but “weak” isn’t the best word for it (if that’s what is meant in the last part of the sentence) .

L565-566: I don’t think the comparison with Honduras is very relevant? Is it just because the point estimate almost the same, or are there other reasons to specifically compare to Honduras? Keep in mind that your 95% CI was 1.3 – 4.3, so it might also be “similar” to other studies above? I also think that this part can be merged with the next paragraph.

We kept this comparative paragraph by specifying at the end of the discussion section the limitations of the study in terms of small sample size knowing that the sample size finally included remains correct as already indicated in explanation n°5.

L557-566 was entirely rephrased in L569-576 in the modified text as following “The prevalence of taeniosis found in this study are quite similar to those previously described (0.1% - 1.4%) in many African countries (Burundi, Congo, Ethiopia, Togo, Zambia, Cameroon, Kenya and Tanzania) and in some endemic countries of Latin America (Peru and Ecuador) using comparable coprological examination. Conversely these prevalence rates could be lower compared to those reported in other African countries (Guinea, Nigeria, Ghana, and Gambia: up to 13%) [12,14,15,54] and those obtained using more sensitive diagnostic tools such as coproantigen ELISA and EITB methods (Zambia, Tanzania, Kenya and Democratic Republic of Congo: up to 23.4% [58–61]”.

L614: I don’t understand the connection between “… Brazil (Figure 3).” and “The description of T. solium…”. Add a sentence to connect both parts or make a new paragraph.

“The description of T. solium…” was a mistake that we should have removed from the initial version.

L629: this sentence suggests that antibodies are lower in people with epilepsy compared to the general population. Rephrase and add more references for the seroprevalence in people with epilepsy.

We have added some references about epilepsy/NCC and the detection of antibodies by EITB and proposed some suggestions that may explain the low prevalence in people with epilepsy compared to the general population in L639-L646 as following in the modified text: “the detection of cysticercosis-specific antibodies during field-based studies could help to identify areas of disease transmission [50] however they tend to over-estimate the prevalence of cysticercosis in sero-epidemiological surveys. Indeed, as antibodies disappear after 1 to 3 years in only 30-40% of seropositive people in endemic countries [72], some seropositivity will reflect past exposure to the parasite. In addition infection in general population could be in any tissue unlike epileptic people with NCC with cysts mainly localized in the Central Nervous System. Lower antibodies related to NCC may also be due to the lower sensitivity of EITB in parenchymal localization with few or calcified cysts [73] [74]”

L634: replace “à” by “to” modified in the text; “weak” is not correct modified with “low” in the modified text

Reviewer #2: Taeniasis/Cysticercosis by taenia solium are diseases which need a multidisciplinary approach to be fought. Authors would consider their study in a more one-health approach and would discuss additional research to be perform in this specific area with the inherent field limitations and highlight in what their result contribute to eradication.

At the end the discussion section in L689-716, we have added a paragraph citing the limitations of this study, its contributions to public health and also a text about the “one health” approach in Madagascar.

Reviewer #3: Minor Revision

The manuscript should review by a native english speaker to improve the quality of the written.

--------------------

<b>Summary and General Comments</b></br></br> Use this section to provide overall comments, discuss strengths/weaknesses of the study, novelty, significance, general execution and scholarship. You may also include additional comments for the author, including concerns about dual publication, research ethics, or publication ethics. If requesting major revision, please articulate the new experiments that are needed.

Reviewer #1: This is an important and interesting study that describes the prevalence of taeniosis and cysticercosis in a remote area in Madagascar. The design and methods are appropriate, but certain aspects should still be clarified (such as the randomisation process and (an estimate) of the total population in these villages, in order to be able to assess the representativeness of the analysis set for the total population, and more details about the questionnaire that was used). The authors should also be more nuanced when drawing conclusions from the data.

Reviewer #2: Although this study give some interesting information upon the realization of a survey upon the use of correct diagnostic test and the sharp genetic identification of parasites from a deprived setting, little is discussed on how about these assays and data could be usefully mobilized to give a dedicated response to the burden of tapeworm infection by Taenia solium in remote areas of Madagascar.

Reviewer #3: This is the first large-scale study in Madagascar to examine the association between potential risk factors measured at the individual-, household-, and village-level and the prevalence of Taenia solium taeniasis/cysticercosis in humans. The authors showed high rates of taeniasis cases affecting all ages in both townships investigated, strongly exposing human and pigs to cysticercosis. This study has a sufficient sample size, well-designed analysis and is quite robust. However, a native english speaker review should improve the quality of writing.
